# Diathesis

**Published:** 1878-04

**Authors:** P. Harold Hayes


### DIATHESIS.

P. HAROLD HAYES, M. D.

My patient J. P. has just come in. It is a warm day in July, with a gentle breeze in the air. He said, "I had to change my flannels two or three times to-day, the weather is so changeable." I find on inquiry that he wears now, July 20th, 1877, two suits of under-flannel, one of which he whips off or on several times a day, as the weather seems to require. He wears an over-coat at breakfast and sometimes at dinner. If you say it is too cold, he instantly agrees with you; and if you say it is too hot, he as instantly accords with you, and will fly to a change in his wardrobe. He says he cannot hear birds sing, roosters crow, dogs bark, pianos played; nay, even the prattle of children distresses him.

Now *diathesis* means a certain condition or habit of the body. This man has the *nervous diathesis.*

Take another case, that of Mrs. R. The family have lately acquired wealth, upon which they set an inordinate value, and she is now tortured with fears that her health is going to fail, and she will not live to enjoy it. She has dwelt upon this thought until her nervous system has become disordered. She goes to one doctor for something to help her digestion, to another for something to help her to sleep, to another for something to help her nerves, and does not know that she has brought on artificially a nervous diathesis or habit of body. Take notice that the suffering in these cases is as real as though it had not been superinduced by a purely morbid attention to self, and over-anxiety as to the health. It is worth while to remark here also, that as in a host of other instances, over-anxiety is sure to defeat itself. And now, if I may moralize just a little, I will remind my readers of the saying of John Stuart Mill, that when the pleasures of life are thought of and pursued as an end, they are quickly felt to be insufficient. But when a great and noble purpose becomes the absorbing thought of the soul, life seems beautiful and desirable.

The nervous diathesis is not only acquired but also transmitted, and we s[illegible]all therefore find many of our patients with whom it is inherited, and in this sense, constitutional. These patients are subject to many forms of purely nervous affections. Spasmodic asthma, spasmodic croup, spasmodic dysmennorrhea, St. Vitus dance, hysteria, neuralgia, and nervous headache, are among the diseases to which this class of patients are especially liable.

There is another diathesis of which I have seen many instances within the year past. It has been well described by M. Hardy, of the St. Louis hospital, Paris, and by him called the *Dartrous Diathesis.* These patients are the subjects of the chronic skin diseases variously known as salt rheum, tetter, and by the profession as eczema, lichen, psoriasis and pityriasis. These are eminently the scaly skin diseases. The subjects of this diathesis often appear to be in fine health, they are active in habits, large eaters usually, and sanguine and vivacious in temperament. The eruptions which characterize this group are characterized by atrocious itching, by a disposition to spread, and by a disposition to relapse from any error in diet, such as the use of salted or cured meats, cheese, spices and coffee. It is remarkable that the dartrous affections I have particularized often have their seat upon the mucus membranes, the internal skin of the body. Thus, bronchitis, asthma, hay asthma, catarrh in its various internal forms, dyspepsia, and many feminine complaints, are purely dartrous affections. It is thus often that these and many other forms of internal diseases are found to be really suppressed skin affections, and when the disease is brought to the surface the internal affection is cured.

There is another diathesis both inherited and acquired, and so common that we scarcely go amiss of it for a single day, and this is the scrofulous diathesis. The subjects of this diathesis are prone, more especially in childhood, to chronic inflammations of the lymphatic system, giving rise to enlarged lymphatic glands, and sometimes to abscess of these glands, especially in the neck. The same form of disease is liable to attack the ends of the long bones, causing what is known as white swelling, or the bodies of the vertebræ, producing ulcerative inflammation, with loss of substance, and if the patient be not promptly and properly treated he is left with a posterior curvature of the spine. In cases of suppressed scrofula, the disease is liable to attack the lungs and ultimately lead to tuberculosis of the lungs, or what is known as scrofulous consumption.

Another diathesis is known as the rheumatic diathesis, and from this habit of body spring the various more purely rheumatic affections, among which are ordinary acute and chronic rheumatism, sciatica and lumbago.

Another diathesis, not very common in this country, but exceedingly common in England, is the gouty diathesis, sometimes called the *lithic acid* diathesis, because the blood is found to be surcharged with lithic acid and lithic acid salts. Gout is much like rheumatism, but a far more serious and painful disease. It attacks by preference the small joints, and when suppressed may attack the heart, lungs or stomach, or give rise to asthma, bronchitis, neuralgia, and disease of the kidneys or bladder.

There is another constitutional condition which fairly deserves to be called a diathesis, and that is the habit of body developed by living long in a malarial atmosphere. So persistent is the effect of malaria, that it is doubtful if, after long residence in a malarious country, the constitution *ever* wholly recovers from its effects. Certain it is that it gives a certain type and character to all the diseases that come after it. They all bear and require in some form or combination quinine and other tonics, and antiperiodics. Bilious diseases, headache, neuralgia, chorea, sciatica and dyspepsia, chronic diarrhœa and jaundice are often of purely malarious origin.

Then there is the hemorrhagic diathesis, the syphilitic diathesis, the corpulent diathesis and the scorbutic diathesis. I will not try to think of any more, for I fancy I hear my readers say already, blessed is the man of no diathesis. Such a man must be a very rare exception. We create or inherit the diathesis, the habit of body, the soil in which our diseases grow.

All really radical treatment strikes for the diathesis; here all precision begins, here all ultimate and positive success has its starting point. Very much of the treatment of chronic diseases is a total failure because it is a treatment of symptoms, by tonics, nervines, expectorants, trying to build up the system on a diseased foundation, while that foundation, the morbid, bodily condition is not treated at all. A gentleman of more than average intelligence asked me to see his wife who he said had lung disease. When I inquired as to the treatment she had received, he said they had just sent to old Dr. James, in Philadelphia, for a cough medicine to cure her cough. When I told him my face was set against all cough mixtures in chronic lung diseases, that they were certain to take away appetite and impair digestion, and they were sure to slowly paralyze the fine bronchial muscles and thereby slowly destroy the power to cough and expectorate, he opened wide his eyes and began to take in a new thought, that it was not the cough as such, but the remoter cause of the cough in the morbid condition of the body and lungs, that we must cure. I know an old lady now over eighty, who in early life had a skin disease suppressed by the kind of management I have indicated, but this was soon followed by rheumatism, and she got this cured—suppressed—only to be followed by the asthma, which had pretty much laid her aside when I found her. As a man who has had the small-pox is forever a changed man as respects that disease, so I believe it to be in our power, by medicines, by climate, by a hygienic system, to entirely change the habit of body as respects disease, so that our patient shall be forever after a changed man and no longer liable to that disease.

Consumption is eminently an in-door disease. The habit of body which creates it is brought on by in-door life. The disease is rife in the manufacturing districts of both Old England and New England, but as you go north in either country, and come into the districts of farming, lumbering, quarrying and pasturage, the disease rapidly diminishes, and finally almost totally disappears. Here out-door life creates a new diathesis of health which keeps at bay forever, nay, destroys forever the scrofulous diathesis upon which consumption depends. So do baths, mineral waters, electricity, wisely used, have the power to change the whole system and create a new and healthy habit of body. Medicines have certainly done the same thing when administered with great skill, in very small doses, and for a very long time together. There has recently been discovered in Cumberland, England, a river which contains a minute quantity of arsenic in solution. This river flows through the village of Whitbeck, and the inhabitants of the village use the water exclusively for every purpose of life. It is so strong of arsenic, however, that it is said that neither ducks nor trout can live in the water, but, nevertheless, the inhabitants and their children are declared, upon the best authority, to be conspicuous for health and long life. The river takes its rise in what is known as the Black Combe mountain, in the lake district, and its waters are perfectly clear, colorless and tasteless. The actual amount of the arsenic is about eight one-*thousandths* of a *grain* to the pint of water. The account further states that lately a railroad was being built near this village, and that the new comers, both men and horses, suffered some inconvenience from the use of the water, but that after a short time the men acquired more than their usual strength, and the horses the sleekness of coat for the production of which arsenic is so remarkable. The particular form of the arsenic is declared, by the same medical authority I have consulted, to be the arsenite of potassa. If nature had intended to set us an example how to use a great constitutional remedy, she could not have directed our attention to one in all respects more striking. And this natural example tells us to use the remedy in minute doses, highly diluted and long continued.

